# Hypokalemic Periodic Paralysis Type 2 Due to SCN4A Val1105Met Mutation: A Case Study

**DOI:** 10.7759/cureus.52063

**Published:** 2024-01-10

**Authors:** Nai-Qing Hu, Jun-Yun Yang, Jin-Lin Lv, Yuan-Zhao Zhu, Li-Hua Li

**Affiliations:** 1 Department of Gerontology, The First Affiliated Hospital of Dali University, Dali, CHN

**Keywords:** genetic testing, potassium supplementation, muscle weakness, scn4a gene, hypokalemic periodic paralysis type 2

## Abstract

Hypokalemic Periodic Paralysis Type 2 (HOKPP2) is a rare autosomal dominant disorder characterized by recurrent episodes of muscle weakness, paralysis, and hypokalemia. In this case report, we present the clinical details of a 49-year-old female diagnosed with HOKPP2. Genetic testing revealed a heterozygous mutation in the Sodium Voltage-Gated Channel Alpha Subunit 4 (*SCN4A)* gene, confirming the diagnosis of HOKPP2. Management strategies, including potassium supplementation and lifestyle modifications, were implemented, resulting in a significant decrease in the frequency of symptomatic episodes. This case highlights the importance of considering HOKPP2 in patients with recurrent muscle weakness, particularly those with a familial history of similar symptoms. Furthermore, it underscores the crucial role of genetic testing in guiding patient management and facilitating genetic counseling.

## Introduction

Hypokalemic Periodic Paralysis Type 2 (HOKPP2) is a rare autosomal dominant disorder characterized by episodes of muscle weakness or paralysis associated with low levels of potassium in the blood [1]. Despite its rarity, it is crucial for clinicians to consider HOKPP2 in the differential diagnosis when patients present with recurrent episodes of muscle weakness, particularly when there is a family history of similar symptoms [2-6]. This case study presents a comprehensive review of a 49-year-old female patient with a history of chest tightness, difficulty in breathing, and general weakness.

## Case presentation

The patient, a 49-year-old woman, has a chronic history of experiencing symptoms such as chest tightness, difficulty in breathing, and generalized weakness for over four decades. She was admitted to the hospital three days prior due to the exacerbation of these aforementioned symptoms. These symptoms typically manifest after two to three hours of heavy physical exertion, such as engaging in strenuous agricultural work. Despite multiple consultations at various hospitals, the etiology of these symptoms remained elusive. Interestingly, the administration of potassium supplementation was observed to alleviate these symptoms. Upon admission to the hospital, the patient presented with severe symptoms including chest tightness, difficulty breathing, significant fatigue, headache, and facial numbness. These symptoms had begun three days earlier, following a period of physical exertion, and at their peak, the patient experienced instability even while standing. Despite receiving unspecified intravenous treatment at a local health clinic, the patient reported no relief in symptoms. The day before admission, the patient began experiencing severe chest tightness and difficulty in breathing, along with significant rigidity and spasms in the extremities. The patient was unable to change position independently and required assistance from family members to regain a normal posture. Occasional abdominal pain was also reported, however, there were no episodes of convulsions, frothing at the mouth, nausea, vomiting, palpitations, or dizziness. During her referral to our hospital for treatment, due to severe symptoms, an emergency electrolyte test at a primary health care center showed a blood potassium level of 2.71 mmol/L, while myocardial enzyme testing was normal. In addition, the patient's family history was significant, with one brother, one sister, and a niece who have experienced similar, albeit less severe and less frequent, symptom episodes.

Investigations

Upon admission, the patient underwent a series of comprehensive medical examinations, including full abdominal and chest CT scans, which did not reveal any abnormalities. The complete blood count showed a white blood cell count of 13.87*10^9^/L, with a neutrophil percentage of 82.3%. The absolute neutrophil count was 11.41*10^9^/L. Further, liver function tests indicated an alanine aminotransferase level of 87 U/L and an aspartate aminotransferase level of 87 U/L. Renal function tests revealed a uric acid level of 441 mmol/L. Notably, the patient's potassium level was low at 3.16 mmol/L, and the amylase level was 27U/L. The patient's blood sodium, chloride, and bicarbonate levels were normal. Moreover, the patient's parathyroid hormone (PTH) and 25-hydroxyvitamin D (25(OH)D) levels were within the normal range, indicating no underlying parathyroid or vitamin D abnormalities.

Additionally, the patient's plasma aldosterone and renin levels were assessed to rule out any adrenal gland disorders, with the results almost falling within the normal range. The patient's creatine kinase levels were also evaluated to check for any muscle damage, and the results were unremarkable (Table 1).

**Table 1:** Key investigations for differential diagnosis PTH, parathyroid hormone; 25(OH)D, 25-hydroxyvitamin D

Items	Value	Reference
Serum PTH	52.56	15-56 pg/mL
Serum 25(OH)D	25.60	30-100 ng/mL
Serum Calcium	2.10	2.15-2.55 mmol/L
Plasma aldosterone (Supine position)	5.71	3.0-23.6 ng/dL
Plasma renin (Supine position)	3.06	2.8-39.9 uIu/mL
Free Triiodothyronine	3.12	3.1-6.8 pmol/L
Free thyroxine	12.5	12-22 pmol/L
Thyroid-stimulating hormone	4.24	0.27-5.6 mIU/L
Creatine kinase	140	24-195 U/L
Urinary pH	5.5	4.5-8.0
24-hour urinary excretion		
Creatinine	144	250-500 mmol/L
Potassium	30.8	36-90 mmol/L
Sodium	130.9	137-257 mmol/L
Chlorine	148.4	170-250 mmol/L
Magnesium	1.16	3-5 mmol/L
Phosphorus	2.80	16.15-42 mmol/L
Calcium	1.16	2.5-7.5 mmol/L

The electrocardiogram upon admission showed ST-T changes in leads V1-V6, and to further rule out coronary heart disease, we underwent cardiac ultrasound and coronary CTA examinations. The cardiac ultrasound and coronary artery CT scan were also normal. Urinalysis was normal, and 24-hour urinary electrolyte levels were within the normal range.

Considering the patient's recurrent symptom episodes and the presence of a similar disease in the family, whole exome sequencing was performed (KingMed Diagnostics, China). This detection was performed on the Illumina sequencing platform. The average sequencing depth of the exon regions and 5bp sequences upstream and downstream of known human genes in the genome was ≥90X, with 98% of the target sequences having a sequencing depth of ≥20X. This detection was established and validated by KingMed Diagnostics. This detection mainly used the GATK software suite (GATK (broadinstitute.org)) for sequencing data analysis. Sequencing fragments were aligned to the UCSC hg19 reference genome (Genome Reference Consortium Human Build 37) using BWA (Burrows-Wheeler Aligner (sourceforge.net)). This detection used the VEP software (Variant Effect Predictor) to annotate variants and screened variants based on genetic disease databases, variant databases, and population-scale sequencing databases such as ClinVar, Online Mendelian Inheritance in Man (OMIM), Human Gene Mutation Database (HGMD®), and Genome Aggregation Database (gnomAD). The sequencing results revealed a heterozygous mutation in the Sodium Voltage-Gated Channel Alpha Subunit 4 (*SCN4A*) gene (chr17: 62025255 NM_000334.4:c.3313G >A (p.Val1105Met)). This genetic finding confirms the diagnosis of HOKPP2.

Management and follow-up

During the patient's hospitalization, she underwent treatment involving potassium supplementation, along with enhanced nutritional support. As a result, the patient's symptoms gradually subsided. The patient was then treated with 20 mg of spironolactone daily. Upon revisiting the outpatient department three weeks post-discharge, the patient's electrolyte levels were found to be completely normal, and there were no episodes of related symptoms (Table 2). 

**Table 2:** Changes in serum potassium levels during treatment and follow-up

Date	Serum potassium	Reference
2023.7.31 (Local health community center)	2.71	3.5-5.5 mmol/L
2023.7.31 (On admission)	3.16	3.5-5.5 mmol/L
2023.8.1	3.30	3.5-5.5 mmol/L
2023.8.4	4.14	3.5-5.5 mmol/L
2023.9.1 (Outpatient department)	4.25	3.5-5.5 mmol/L

Two months after discharge, during a telephonic follow-up, it was noted that aside from slightly poor appetite, the patient did not experience any significant episodes of symptoms such as chest tightness, shortness of breath, fatigue, or difficulty in breathing. The patient's family was advised to undergo genetic testing; however, they declined further examination.

## Discussion

The *SCN4A* gene encodes the alpha subunit of the voltage-gated sodium channel, which is predominantly expressed in skeletal muscles. This channel is responsible for the initiation and propagation of action potentials in skeletal muscle, and mutations in this gene can lead to alterations in muscle excitability. Mutations in *SCN4A *have been implicated in a wide spectrum of neuromuscular disorders with variable onset, ranging from a rare form of congenital myasthenic syndrome to both hypokalemic and hyperkalemic forms of periodic paralysis, paramyotonia congenita, and even laryngospasm [7].

The specific mutation in the *SCN4A* gene discussed here, c.3313G>A (p.Val1105Met), results in the substitution of a valine residue with a methionine at position 1105 of the protein. This position is located in the fourth domain of the sodium channel, which is critical for voltage sensing and channel gating. The *SCN4A* Val1105Met mutation is relatively rare and has only been detected in one case of sudden death with unknown causes in the past [4].

The valine at position 1105 is highly conserved across species, suggesting its importance in the function of the sodium channel. The substitution of this valine with methionine could potentially alter the structure and function of the sodium channel. According to the gnomAD database, this particular variant has been observed in five individuals in a heterozygous state, with no reported cases of homozygosity. Furthermore, the literature review has associated this variant with a single instance of unexplained sudden death [4]. Currently, the clinical relevance of this mutation remains undetermined. Nevertheless, to gain deeper insights into its potential functional consequences, we concur that further functional studies or a comprehensive familial investigation are warranted.

## Conclusions

This case underscores the importance of considering a diagnosis of HOKPP2 in patients presenting with recurrent episodes of muscle weakness, particularly when there is a family history of similar symptoms. Genetic testing can provide a definitive diagnosis, guiding management, and genetic counseling. Despite the reluctance of other family members to undergo genetic testing, the patient's clinical presentation and whole exome sequencing helped rule out other diseases. The patient's follow-up revealed no significant symptom occurrence in the past two months, although she reported poor appetite.
